# Malignant Leydig Cell Tumor in Elderly Complete Androgen Insensitivity Patient: A Case Report

**DOI:** 10.31729/jnma.4206

**Published:** 2019-04-30

**Authors:** Sundar Shrestha, Niroj Banepali, Rakesh Sthapit, Dipesh Agrawal

**Affiliations:** 1 Department of General Surgery, Bir hospital, Kathmandu, Nepal

**Keywords:** *complete androgen insensitivity syndrome*, *leydig cell tumour*, *testicular feminization*

## Abstract

There are various causes of primary amenorrhea in phenotypically females such as, complete androgen insensitivity syndrome, pure gonadal dysgenesis, 17b-hydroxysteroid dehydrogenase deficiency, or mixed gonadal dysgenesis. Primary amenorrhea in a phenotypically female is commonly encountered in Androgen Insensitivity Syndrome. In patients with Androgen Insensitivity Syndrome, with intraabdominal testis there is high chances of developing testicular tumour, among them Sertoli cell tumour and seminoma being the most common types. Leydig cell tumour in androgen insensitivity syndrome, is very rare and malignant leydig cell tumour is even further rarer. There are few cases reported in the literature of malignant leydig cell tumour with complete androgen insensitivity. Here we are reporting a case of 65 years married elderly patient with malignant leydig cell tumour with complete androgen insensitivity syndrome.

## INTRODUCTION

Androgen Insensitivity Syndrome (AIS) or testicular feminization is a sex-linked recessive inherited disorder caused by a mutation of the androgen receptor gene located at Xq11-q12J Patients with AIS have abnormal descend of the testes, histologically revealing solid immature tubules and markedly decreased or absent germ cells. The gene (containing 8 exons) for the androgen receptors is located on the long arm of X chromosome.^2^ These patients are phenotypically, anatomically and socially female but have a gonad and male genetic composition. Testicular tumors can develop in patients with AIS and that the risk of gonadal malignancies increases as the age increases. Although AIS is diagnosed at puberty in primary amenorrhea with phenotypically female patients, is occasionally diagnosed in an older population also. Complete Androgen Insensitivity Syndrome (CAIS) is associated with abnormal testicular development with an increased risk of germcell malignancy. As the age advances the risk of malignancy increases.^3^ Sertoli-Leydig cell tumours are very rare gonadal tumours, majority being benign.

There are reports of various types of tumours in AIS patients. Though Leydig cell tumour is the most common type of pure sex cord-stromal tumour of the testis in the general population, Leydig cell tumour in patients with AIS is extremely rare. We hereby reporting a case of malignant Leydig cell tumour in a 65-year-old patient with complete AIS.

## CASE REPORT

A 65 years old patient was referred to Bir Hospital, Kathmandu Nepal from the local hospital in Hetauda, Nepal with huge inguino-scrotal swellings, which became tense and painful for last 1 month after the attempt of aspiration from the swelling. Patient is married for the last 35 years, though having no children and with primary amenorrhea but has not consulted doctors irrespective of the symptoms. On physical examination, patient is 165 cm tall and 55 kg weight with normal breast and sparse axillary and pubic hair development. The external genitalia appeared to be those of a normal female. The vagina was shortened and blind. There was no uterine cervix observed. There was no physical evidence of virilisation.

Routine haematological and biochemical investigations were within normal limits. Ultrasound examinations of the abdomen, pelvis and perineum revealed bilateral testis. There was a mixed echoic lesion with mixed vascularity noted in bilateral scrotal sac. However, defect was not appreciated likely inguinoscrotal hernia. Patient has two sisters and two brothers; none of them had similar abnormalities and they are married with children. Hormonal profile and tumour markers for the patient evaluated showing: serum LDH/Alpha Feto-protein/ serum beta HCG: WNL, testosterone: 0.92 nmol/L [Male: 2.41–21.6 nmol/L; Female: 0.1982.67 nmol/L], progesterone: 0.22 ngm/ml [Male: <1 ngm/ml; Female: 0.27–25.6 ngm/ml], estradiol: 26.35 pgm/ml [Post-menopausal female: 5.37–38.4 pgm/ml; Male: 5.37–68.5 pgm/ml].

CT scan abdomen and pelvis showed undescended right testis, heterogeneous enhancing enlarged left testis likely due to inflammatory causes- orchiepididymitis, rudimentary prostate.

Cytogenetic analysis demonstrated a male karyotype (46,XY). Based on these data, patient was diagnosed as Complete Androgen Insensitivity Syndrome (CAIS).

Patient was taken for surgical exploration, which revealed large 25 cm × 20 cm size swelling with haemorrhagic fluids in the sac with firm irregular mass in sac involving the left testis. Right testis was within the right inguinal canal.

**Figure 1A, B:**
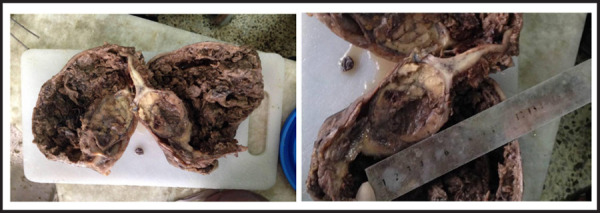
Tan white mass with multiple areas of hemorrhage with some normal appearing testicular tissue.

**Figure 2A, B:**
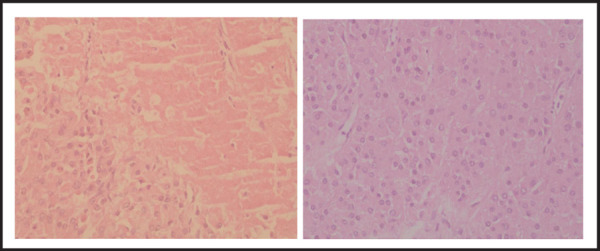
Microscopic pictures. Uniform atypical cells arranged in diffuse sheets, abundant eosinophilic cytoplasm with distinct cell borders, necrosis and occasional mitotic figures.

The final pathological diagnosis of the tumour was malignant leydig cell tumour of the left testis. The extirpated left gonadal tumors was brown coloured, single piece of capsulated globular tissue measuring 17cm × 1.5 cm × 8 cm, testis: 7.5 cm × 6 cm × 3.5 cm. Cut section showing, tan white mass with areas of haemorrhages measuring 7 cm × 4 cm × 3 cm (Figure 1 A, B). Histologically showing, uniform atypical cells arranged in diffuse sheets, abundant eosinophilic cytoplasm with distinct cell borders, necrosis and occasional mitotic figures (Figure 2 A, B). Therefore, the tumour was diagnosed as malignant. The tumour size and gross appearance with massive necrosis and haemorrhage also supported the malignant nature of the tumour. However, there was no microscopic invasion of tumour cells to the lymphatics or blood vessels.

Post operatives days were uneventful and the patient was discharged on the tenth postoperative day. Patient was asked to follow up on the subsequent OPD visits for further evaluation and the need for adjuvant chemotherapy but lost on follow up there after.

## DISCUSSION

CAIS is a rare pathologic condition, incidence ranging from 1/60,000 to 1/20,000 births.^4^ It occurs due to a defect in androgen receptor function, causing peripheral androgen resistance. It is characterized by a normal female phenotype however with a male karyotype. There is presence of female breast development, although no axillary or pubic hairs and absence of mullerian duct derivatives. The external genitalia appear normal with usually shortened and blind end varying length of the vagina. The cervix and uterus are absent. The undescended testes may lie along the normal course of testicular descent like in abdominal cavity, the inguinal canal or the labia majora.

The estimated risk of malignancy the testis is 5–10% until 25 years of age and increases up to reach 33% at age 50 years.^5^ These testes should be removed by either laparotomy or preferably using laparoscopy in case of intra-abdominal testes. Leydig cell tumour, very rare in patients with AIS, is though the most common type of testicular pure sex cord-stromal tumour in the general population. Commonly seen tumours in AIS are Sertoli cell tumour and germ cell tumours. Rutgers and Scully has reported one case of Leydig cell tumour in patients with AIS in the series of 43 cases.^6^ They reported 1.5 cm in diameter Leydig cell tumour without crystals of Reinke in a 49-year-old patient. In the largest series of Leydig cell tumours of the testis by Kim et al, reported the prognosis and characteristics of this malignant tumor.^7^ They were followed-up from 2 months to 22 years (average 4 years). When they compared survivors and non-survivors, showed that the factors associated with a decreased survival were increasing patient age, larger tumour size, infiltrative margin, spread beyond the testis, invasion to blood vessels or lymphatics, greater degree of cellular atypia, necrosis, and a higher mitotic rate. Our case had four out of eight above features described.

Leydig cell tumour is rare in-patient with AIS. Malignant Leydig cell tumour is further rare. Our case represents the identification of a rare tumour in a minority group of people in resource-limited country like in Nepal, which is genetically and histologically proven.
